# Four Seasons for Schwann Cell Biology, Revisiting Key Periods: Development, Homeostasis, Repair, and Aging

**DOI:** 10.3390/biom11121887

**Published:** 2021-12-15

**Authors:** Gabriela Sardella-Silva, Bruno Siqueira Mietto, Victor Túlio Ribeiro-Resende

**Affiliations:** 1 Instituto de Biofísica Carlos Chagas Filho, Universidade Federal do Rio de Janeiro, Rio de Janeiro 21941-902, RJ, Brazil; gabrielasardella@biof.ufrj.br; 2 Núcleo Multidisciplinar de Pesquisa em Biologia (Numpex-Bio), Campus de Duque de Caxias Geraldo Guerra Cidade, Universidade Federal do Rio de Janeiro, Duque de Caxias 25255-030, RJ, Brazil; 3 Instituto de Ciências Biológicas, Universidade Federal de Juiz de Fora, Juiz de Fora 36036-900, MG, Brazil; bruno.mietto@ufjf.edu.br

**Keywords:** Schwann cells, peripheral nervous system, development, homeostasis, regeneration

## Abstract

Like the seasons of the year, all natural things happen in stages, going through adaptations when challenged, and Schwann cells are a great example of that. During maturation, these cells regulate several steps in peripheral nervous system development. The Spring of the cell means the rise and bloom through organized stages defined by time-dependent regulation of factors and microenvironmental influences. Once matured, the Summer of the cell begins: a high energy stage focused on maintaining adult homeostasis. The Schwann cell provides many neuron-glia communications resulting in the maintenance of synapses. In the peripheral nervous system, Schwann cells are pivotal after injuries, balancing degeneration and regeneration, similarly to when Autumn comes. Their ability to acquire a repair phenotype brings the potential to reconnect axons to targets and regain function. Finally, Schwann cells age, not only by growing old, but also by imposed environmental cues, like loss of function induced by pathologies. The Winter of the cell presents as reduced activity, especially regarding their role in repair; this reflects on the regenerative potential of older/less healthy individuals. This review gathers essential information about Schwann cells in different stages, summarizing important participation of this intriguing cell in many functions throughout its lifetime.

## 1. Introduction

Schwann cells are the principal glia of the peripheral nervous system (PNS), which virtually orchestrates most, if not all, pathophysiological processes that develop in the nerve (as reviewed in [1]). Schwann cells outnumber the other non-neuronal cells that populate the nerve (i.e., macrophages and fibroblasts) and are uniquely positioned side-by-side along the axonal projections, making these cells a great partner for neuronal physiology and nerve tissue homeostasis (as reviewed in [2]). Regarding peripheral neuropathies, the success of nerve regeneration is attributed to changes in Schwann cell responses, including their ability to secrete inflammatory mediators, to clear cellular and myelin debris and to produce a broad range of regenerative factors, making modified Schwann cells great orchestrators of nerve repair [3,4,5]. In addition to their role in the injured microenvironment, several reports also identify mutual communication between Schwann cells and axons, with the transferring of cargos and metabolites from glia to the axon, which are crucial for the maintenance of axonal integrity and function [6,7], (for review, see [8]). Finally, all this intense biological activity has natural cell consequences generated by mitochondria-derived oxidative stress. The cells acquiring their aging phenotype lose the capacity to react and orchestrate most of their abilities to support homeostasis, degeneration, and regeneration. The importance of Schwann cells to peripheral nerve function is unquestionable, and for this matter, this review will focus on the four key periods of nerve biology (development, homeostasis, repair, and senescence) and how Schwann cells modulate and are influenced by these phases. Finally, in this review, we find that Schwann cell biology could be philosophically correlated with year seasons because, in this context, the energy levels are very similar. Therefore, this allows us to analyze scientific data about Schwann cells and generate a dynamic text on its biology.

## 2. Spring for Schwann Cells: The Development

Spring is the season in which all beauties naturally arise, starting from an innate genetic program that is accompanied by and influenced by “the practicing”. These complementary intricate interactions between nature and nurture, applied to all cells over time throughout body development, generate distinct cellular specializations, including those in the peripheral nervous system (PNS). Therefore, the appearance and development of all cells in the PNS recollect the spring season when a great cell biology orchestra is playing very well tuned and synchronized processes to define the fate and function of these cells.

Schwann cells, in addition to melanocytes, dorsal root ganglion (DRG) neurons, autonomic neurons, and chromaffin cells, all originate from neural crest cells [9,10,11,12,13], whose migratory pattern is determined by bone morphogenetic proteins (BMPs) [14,15], Wnt, and FGF signaling [16,17,18,19,20,21,22]. The glial fate starts to be established once the migration initiates, as they migrate ventrally alongside autonomic neurons and chromaffin cells, while melanocytes and DRG neurons migrate by a lateral pathway [12,13]. Furthermore, the melanocytic lineage expresses c-Kit receptors, while it has been shown that the neural-fate is associated with TrkC expression on these cells [10]. After migrating and directing to the glial lineage, the neural crest cells undergo an organized and time-specific differentiation process that goes from the embryonic to the post-natal stages. The main signaling pathway seems to be the Notch-Delta/neuregulin/endothelin-3 [23,24,25,26,27,28], which acts by orchestrating the expression of several transcription factors, such as SOX10, a transcriptional factor that marks the transition from neural crest cell to a committed glial precursor [29,30]. At about E14 (rats) and E12.5 (mouse), these cells turn into Schwann cell precursors at the equivalent time when the nerve develops, associating themselves with the first growing axons. When compared to neural crest cells, Schwann cells start expressing several different molecules, such as Cadherin 19 (Cad19) and GAP43, stop being associated with and dependent on the extracellular matrix (ECM) and begin to depend on the survival factor secreted by the growing axon, Neuregulin 1 (NRG1), via activation of receptors ErbB2/ErbB3 [11,24,26,31,32]. These precursors are important for the fasciculation and long-term survival of both sensorial and motor neurons, through the secretion of GDNF and NT3 [33,34,35,36]. These cells rapidly differentiate into the next phenotype, the immature Schwann cell (around E17 in rats), which lasts up until birth when the cells acquire the terminal differentiation. As they go from precursors to immature cells, they progressively express S100B protein, which, from this point on, remains expressed even on terminally differentiated cells [11]. They also become independent from the axonal survival signaling, as they stop responding to NRG1 and start an autocrine survival circuit that includes a broad range of neurotrophic factors, including FGF, IGF, ET, PDGF, and NT3 [37,38,39]. This ability of secreting neurotrophins, initiated during Schwann cells development, will print out a molecular signature on these cells, allowing them to recapitulate part of this genetic program when the nerve is perturbed by an injury (as reviewed in [1]).

At this stage, the basal lamina becomes organized, and the Schwann cells participate in the arteriogenesis signaling alongside neurons by secreting VEGF [40,41], promote the differentiation of the connective tissue by the expression of Dhh [42], and organize the radial sorting, a crucial step to the correct myelination of the PNS [43]. On this radial sorting event, the immature Schwann cells group axons and then proceed to proliferate and infiltrate through these groups, resulting in events known as fasciculations, when large caliber axons are segregated from the bundles that later become non-myelinated Remak bundles. This segregation is the cause of the well-known 1:1 ratio observed in myelinated axons. Thereafter, the immature Schwann cells stop their proliferation and begin the production of basal lamina in the process of defasciculation [43,44,45]. The signaling for the proper sorting organization derives from components found in the basal lamina, as well as from the axons, including Wnt and R-spondins signaling pathways [45,46,47,48,49,50,51]. After birth, this radial sorting continues up until a few post-natal days [43].

After birth, the myelination phenotype of the nerve is initiated in a process driven by signals provided by axons that promote the immature Schwann cells to a myelinating or non-myelinating phenotype, through the biochemical marker of axonal caliber NRG1 type III. Large caliber axons express high levels of type III NRG1 on their surfaces, activating ErbB2/ErbB3 receptors present on the Schwann cell membranes in a way that overcomes the minimum threshold required for myelination induction [52]. After surpassing the threshold, the more NRG1 is expressed, the thicker the myelin sheath will be [53]. However, small caliber fibers express low levels of NRG1 and do not reach this minimum signaling threshold necessary to trigger Schwann cells to enter the myelination program. In fact, these axonal segments are organized in groups, assembled by one non-myelinating Schwann cell, forming the known Remak bundles by the end of the radial sorting period, in rodents [52].

The external signaling to a myelinating phenotype leads to the expression of many molecular regulators. The transcriptional factor SOX10 acts in synergy with factors Tst-1/Oct-6/SCIP (also called Pou3f1) and Brn2 (also called Pou3f2), expressed in immature cells, by bounding to the myelinating Schwann cell element (MSE) that directs expression of another transcriptional factor, Krox20 [54,55,56]. Another pathway leads to this same terminal effect, as NRG1 has been shown to activate the PLC-γ pathway, dephosphorylating the transcriptional factors NFATc3 and NFATc4, the latter also being capable of synergically interacting with SOX10, ending in Krox20 activation [57]. NRG1 also regulates expression of Krox20 by phosphorylating Yin Yang 1, a factor that also binds to the MSE [58]. Krox20, also known as Egr2, is the well-stablished main regulator of myelination promotion and maintenance, as it needs to be constantly expressed [59,60,61].

After the long spring and intricate cell-cell and cell-matrix interactions, finally, the nerve is ready to perform its function by having Schwann well-formed and positioned cells support the nerve in health and disease.

## 3. Summer for Schwann Cells: Adult Homeostasis

The adult stage of the nerve is a period of high energy flow with great part coming from Schwann cell metabolism in its mature phenotype, permitting the functionality of the adult peripheral nerve. In addition to their role in being wrapped around axons, Schwann cells are also responsible for energy storage and delivery, providing the necessary metabolic coupling for neuronal function and homeostasis (as reviewed in [62]). This neuron-Schwann cell bidirectional communication is a highly energetic rout, such as the summer period.

Schwann cells are key players on the maintenance of the PNS homeostasis (as reviewed in [63]). It has been demonstrated that Schwann cells, as well as their precursors, secrete neurotrophic factors, such as GDNF and NT3, which are crucial for the survival of both sensitive and motor neurons [33,35]. Myelinating Schwann cells are involved in many processes, such as the phosphorylation of axonal neurofilaments [64], and they also express the myelin-associated protein (MAG) that regulates axonal caliber [65]. One important regulatory role of these cells is the formation of the nodes of Ranvier, by inducting nodal membrane molecular specialization processes, such as the clustering of sodium channels. Surprisingly, this event occurs in a contact independent manner, through the secretion of both channel-forming factors, as well as surface molecules that restrict the spatial distribution of these channels [66,67].

Similar to what happens in neuronal and other glial types, besides the myelinating/non-myelinating profiles, it has been shown that myelinating Schwann cells present different patterns of growth factor production accordingly to sensory or motor association preference and in a central-peripheral axis manner, further dividing them in subtypes [68,69].

While oligodendrocytes are important to the metabolic support of central axons by offering substrates that are glycolysis-derived, but not originated from mitochondrial respiration [70,71], Schwann cells participate in the metabolic support of peripheral axons in a mitochondrial dependent pathway [72]. Mitochondrial disruption has been indicated to cause demyelination, altered stress response, neuroinflammation, and axon degeneration [6,73] (for review, see [8]), which can be linked to the role of Schwann cell metabolism over pathologies such as the diabetic neuropathy [74]. Furthermore, the requirement of iron transfer from Schwann cells to axonal mitochondria for proper axonal physiology and regeneration was recently shwon [75]. In fact, a molecular pathway involved in the axon metabolic maintenance by Schwann cells was demonstrated to be the liver kinase B1 (LKB1) and its downstream target AMP activated protein kinase, LKB1-AMPK, which is altered in many neuropathologies and aging [76].

The summertime for Schwann cells based on the description above can be sustained throughout the adult life, but as demonstrated over the last few decades, there are multiple aspects that can trigger a summer fading out and bringing up these cells to the next season, where pathogens, injuries, or metabolic diseases will trigger changes in Schwann cells phenotypes, adapting peripheral nerves to the degenerative and regenerative process. This is Autumn.

## 4. Autumn for Schwann Cells: The Degeneration for a Potential Regeneration

Unlike what is observed in the injured CNS, the PNS has great regenerative potential, even though it may occur in an incomplete or non-functional manner [77] (for review, see [78]). This re-growing potential is of great importance, as the PNS per se does not have bone protection, such as its central counterpart, leaving the peripheral tissue under severe risk, ranging from either external or internal traumatic origins, such as infections, genetic, and metabolic disorders. Those misfortunes somehow bring the autumn season to Schwann cells, and their plasticity is fundamental for proper degenerative processing, with positive consequences for regenerative ability.

Adult Schwann cells have an autocrine survival circuit that is of vital importance during the occurrence of injury [37,38,39]. After nerve injury and subsequent distal axon degeneration, these terminally differentiated cells signaled by the loss of axonal contact become part of the regenerative program of the PNS, through the ability of shifting into a regenerative immature-like phenotype, the repair Schwann cell [79,80]. After axonal traumatic injury, both CNS and PNS go through a degenerative process named Wallerian degeneration [81], which is markedly slow in the CNS [82,83,84,85], affecting the regeneration potential of central neurons. This degeneration on the mammal PNS is characterized by a sequence of events that go from 36 h to 7–14 days post injury [83,86,87], while on the CNS, it can take anywhere between months and years [88,89]. In the PNS Wallerian degeneration process, initial axonal degeneration is followed by myelin degradation. In addition to the contribution of macrophages to the overall removal of myelin and axonal debris, Schwann cells ingest myelin ovoids by a broad range of receptor-mediated phagocytosis [90,91] and by activating the intracellular autophagic myelin clearance [92,93]. Macrophage recruitment, activation, and clearance of tissue debris is also driven by Schwann cells, as they secrete inflammatory cytokines and chemokines that attract the hematogenous macrophages and induce proliferation of resident macrophages [3,94]. These Schwann cells lacking axonal contact also proliferate transiently and transdifferentiate into the repair phenotype [95]. The transcription factor c-Jun is considered the key orchestrator of the repair phenotype differentiation as it regulates a series of regeneration-related genes for different Wallerian degeneration steps: myelin clearance, neuronal survival, formation of Büngner bands, and axonal regrowth [4]. c-Jun is also a known negative regulator of the key myelin transcription factor Krox20 [96], as well as other myelin proteins, such as MBP and MPZ. While signaling the downregulation of myelination genes and myelin-forming elements, such as Krox20, MPZ, and MBP, it also upregulates immature markers, such as NCAM and p75NTR, indicating the dedifferentiated character of these cells [4]. To support the regeneration process, c-Jun also controls the known post-injury secretion of support growth factors, such as BDNF and GDNF [4], with other growth factors, such NGF, VEGF, and IGF, which are also being upregulated. Interestingly, Schwann cells from different fibers not only differ in their growth factor expression levels on homeostasis but also after injury, as was shown by Hoke et al. (2006) [69] and Brushart et al. (2013) [68]. Finally, these cells proliferate and shift their morphology to an elongated shape that is three-fold larger than the average mature Schwann cell. This is fundamental to the formation of the Büngner bands that act as a physical support, like a rail, to the reconnection of axons to their target tissues [79].

In physiological conditions, CNS and PNS axons are myelinated by different glial cells. While Schwann cells myelinate peripheral axons, the same axonal ensheathment is stablished by oligodendrocytes over the CNS [97,98]. Other than sharing this myelinating property, these two cells have different origins, morphology, and molecular profile [99]. These properties confer a vastly different response when it comes to repair. Different from the Schwann cell, oligodendrocytes do not help the myelin clearance process, nor do they support axons reconnecting to their lost targets. Due to the loss of axonal contact in degeneration, these cells enter an apoptotic or quiescent state. Therefore, the presence of myelin debris and its growth-inhibitor molecules, alongside the absence of axonal guidance, provide a very unfavorable environment to axonal regrowth [100,101,102,103]. However, endogenous Schwann cells were found inside the CNS both in animal models [104] and humans [105], associated as an occurrence (mostly on the spinal cord) after conditions of various origins that result in demyelination [106,107,108,109] or axonal injury [110]. The most common thought was that these cells migrate from the PNS into the CNS over pathologies and lesions to offer a similar regenerative role exhibited on the PNS. This opened a series of studies evidencing the transgression, remyelinating, and regenerating capacities of these cells in many different pathologies and conditions [106,107,108,109,110,111,112,113,114,115,116,117,118,119,120], as well as their regenerative-remyelinating potential after exogenous addition [121,122,123,124,125,126,127].

Although this PNS-CNS migration does happen, these PNS Schwann cells have been seen to colonize the more external portions of the spinal cord, on areas closer to the CNS-PNS interface [128], indicating that PNS-Schwann cell migration within the CNS is somehow limited. Recently, it has been shown through ectopically-placed PNS-Schwann cells that migration in the CNS is mediated by Eph/ephrin signaling, using blood vessels as a preferential substrate [129]. The Eph/ephrin interactions guide the migration because they can work both as positive or negative adhesion signals in many cell-cell adhesion events [130], and, in this scenario, it works by not only favoring the association to the blood vessels but also by the avoidance of CNS myelin. CNS myelin contains EphrinB3, an axon-growth inhibitor molecule, which acts through Schwann cell Eph receptors, EphA4 and EphB6, causing reduced adhesion and cell processes formation. However, the same EphrinB3, through interactions with integrinβ1, can improve Schwann cell adhesion to the perivascular fibronectin. Myelin was shown to upregulate expression of integrinβ1 and repel Schwann cells through its myelin-associated glycoprotein (MAG) [129]. Moreover, it is well known that MAG is also an anti-axon regeneration protein that binds to Schwann cells with high-specificity and promotes p75 cleavage, lacking Schwann cell migration, with consequent cell death [131].

As mentioned before, PNS Schwann cells have been identified mostly on the outer regions of lesions, nearer to PNS connections [128]. Gilmore et al. (1982) [115] observed time-dependent differences in Schwann cell distribution and myelin maturation over the lesion area. They encountered Schwann cell and mature myelin firstly on the outer portions, changing patterns in a dorsal-ventral direction. Blakemore et al. (1987) [132] suggested that these cells could either be Schwann cells or cells capable of becoming Schwann cells, once the demyelinated spinal cord presented Schwann cell remyelination after injections of CNS cells.

The remaining question is: where do these Schwann cells encountered in the center of CNS lesion sites come from? Recently, it has been shown that, surprisingly, these cells have a different origin. According to studies on both spinal cord injury [128] and demyelination [133], Schwann cells in the CNS can also be derived from oligodendrocyte precursor cells (OPCs), a fact observed using fate-mapping studies on the OPCs marker platelet-derived growth factor receptor α (PDGFRα). Thus, besides forming remyelinating oligodendrocytes, OPCs originate most of the remyelinating Schwann cells, producing functioning cells with positive peripheral myelin marker P0 and characteristic morphology [128]. Interestingly, so far, these OPC-derived Schwann cells have been evidenced to retain two features of OPCs: the potassium channel current and O4 and A2B5 ganglioside higher expression. These cells also seem to present lower expression of GFAP when compared to its peripheral relative [134]. The mechanisms involved in this OPC-derived Schwann cell differentiation have been studied and might be due to the disbalance between the BMP/Wnt pathway and its antagonist, Sostc1. The proposed mechanism is that demyelination would cause upregulation of BMP4 in OPCs over the loss of homeostasis, which, in the absence of Sostdc1, expressed by reactive astrocytes, leads them to differentiate into Schwann cells [135]. Astrocytes also regulate the maintenance of CNS specificity, balancing oligodendrocyte and Schwann cell differentiation [136]. The molecular mechanisms for this balance seems to work through STAT3 signaling (as reviewed in [137]). Importantly, Schwann cells not only promote the remyelination process itself but allow later oligodendrocyte remyelination [118].

In addition to CNS myelin, astrocytes provide another barrier to Schwann cell infiltration. Firstly, astrocytes constitute the glia limitans (GL) that delimitate CNS and PNS compartments (as reviewed in [138]), therefore limiting PNS-derived Schwann cells entry. They also form a barrier over blood vessels [139]. CNS and PNS-Schwann cell migration and remyelination can only occur in astrocyte-free zones, provided either by astrocyte death, following injury or disease [109], or by irradiation or chemical ablation [106,114,115,120]. In fact, CNS lesion sites are knowingly delimitated by glial scar [140,141,142,143,144,145], constituted of reactive astrocytes, which could both possibly break the GL barrier [119] and delimitate the area so that Schwann cells do not spread beyond the lesion site [146]. This astrocyte barrier blocks the passage of Schwann cells but also the complete regrowth of axons, therefore challenging regeneration [141,142]. This barrier is not only physical but also molecular, having been linked to NCAM [147,148,149,150], PI3K-Akt signaling pathway through the mediation of neuregulins, and FGF [151], interaction between astrocytes EphrinAs and Schwann cell receptors EphA4 and EphA7 [152], and the presence of chondroitin-sulfate proteoglycans [153,154,155,156].

The first three seasons naturally require intense mitochondrial activity to provide enough energy to support such a complex and well-orchestrated biological process. It has been well understood that there are age-related drawbacks thar are linked to the accumulated levels of reactive oxygen species (ROS).

## 5. Winter for Schwann Cells: Aging

The aging of Schwann cells is not only related to the lifetime of cells from an elderly animal. This may be a winter in the right period, remarkably anticipated by a hostile modified environment or genetic/infected disorder. Altered metabolic patterns, reduced adaptation to stress, accumulation of damaged proteins, lipids, and DNA, as well as pathobiological and traumatic aspects, can accelerate cellular senescence and neurodegeneration, disturbing Schwann cell ability to support distinct aspects of peripheral nerve homeostasis, degeneration, and regeneration.

It is no novelty that the process of aging brings to the whole body, including the peripheral nerves, complex morphophysiological alterations. While it has been shown that many of these alterations include Schwann cells, the molecular mechanisms involved are still poorly elucidated. Even on physiological conditions, aging has been shown to affect nerve fibers with reduced function. This age-related deficit seems to be related to loss of oxidative damage protection and/or repair [157], an altered transcriptional map, especially on lipid metabolism and immune response [158], and loss of the heat shock protein, alphaB-crystallin (αBC) [159].

However, what has well-demonstrated is how aging affects the regenerative potential of the PNS by mainly altering the Schwann cell and its plasticity capacity, alongside modifying the connective tissue and macrophages [160]. Slow, delayed, and/or incomplete regeneration of aging injured nerves were thought to be due to the loss of the axon intrinsic regeneration capacities over aging. However, this poor regenerative capacity is associated with the unfavorable environment offered by aged Schwann cells, such as reduced debris clearance and decreased production of neurotrophic and growth factors [160], once those aged axons regrow normally in favorable conditions [160,161]. In fact, aging changes the normal occurrence of Wallerian degeneration by altering the regular molecular regeneration program of Schwann cells, therefore affecting multiple aspects of their differentiation towards the repair phenotype. When compared to Schwann cells from young, injured nerves, aged Schwann cells fail to upregulate pro-regenerative genes related to growth factors and mitosis; they also failed to downregulate myelin genes, an important step to enter the regenerative process [160]. The repair-associated transcription factor c-Jun shows a different acute regulation pattern in aged animals, which is aligned to the similarities seen in the transcriptional profiles of aged and c-Jun KO mice after injury [91,160,162]. This different environment during aging also affects the myelin clearance, not only by the Schwann cells but also because it alters the macrophage attraction and, therefore, immunomodulation [160,163,164]. Additionally, not only do Schwann cells age, but also the macrophage population, and it has been seen that both senescent Schwann cells and macrophages present reduced phagocytic capacities [160,164].

**Figure 1:**
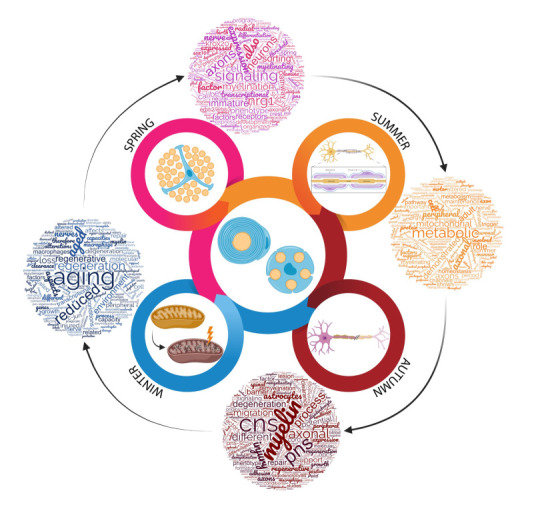
Dynamic art correlating the Schwann cell biological stages with the four seasons.

When a full year equivalent of a Schwann cell’s life has passed, it is unquestionable that this cell is not only a member of the peripheral nervous system’s philharmonic, but, in fact, it is its own conductor (Figure 1). Understanding it as a whole, all its potential and all its limitations, is an open door to the exploration of its many possibilities as a tool for regenerative medicine.

## 6. Closing Remarks

Schwann cells are vital for peripheral nerve development, homeostasis, and multiple degenerative and pro-regenerative responses. The full understanding of the role of Schwann cells in peripheral nerve health, senescence, and disease is an exciting field for deciphering how this glia underlies nerve integrity and pathology. This knowledge is necessary for developing effective strategies to restore the homeostasis of peripheral nerves, as well as its regenerative capacity after any disturbance. In particular, it becomes a great challenge when tissue segments are lost. Combined strategies for nerve regeneration using Schwann cells have been successfully developed over the last four decades, but unfortunately come with partial regain of function and are often followed by drawbacks, such as neuropathic pain or paraparesis. The comprehension of Schwann cell biology before or at least with the development of a new regenerative strategy is a careful scientific way to understand, step-by-step, the evolution of nerve tissue replacement and functional recovery.

## Data Availability

Not available.
